# Virtual Reality for Workplace Violence Training of Health Care Workers: Pilot Mixed Methods Usability Study

**DOI:** 10.2196/70817

**Published:** 2025-09-15

**Authors:** Daniel Isaiah Jackson, Thipkanok Wongphothiphan, John Luna, Tensing Maa, Mary A Fristad, Yungui Huang, Brittany Schaffner, Jennifer Reese, Jason Wheeler, Brandon Abbott, Emre Sezgin

**Affiliations:** 1 Center for Biobehavioral Health, Abigail Wexner Research Institute, Nationwide Children's Hospital, 431 S 18th Street, Columbus, OH, 43205, United States, +1 6147222000; 2 IT Research and Innovation, Abigail Wexner Research Institute, Nationwide Children's Hospital, Columbus, OH, United States; 3 Center for Clinical Excellence, Nationwide Children's Hospital, Columbus, OH, United States; 4 College of Medicine, The Ohio State University, Columbus, OH, United States; 5 Division of Child and Family Psychiatry, Nationwide Children's Hospital, Columbus, OH, United States; 6 Department of Psychiatry and Behavioral Health, The Ohio State University, Columbus, OH, United States

**Keywords:** virtual reality, usability, interprofessional, frontline health care staff, workplace violence, mixed methods

## Abstract

**Background:**

Workplace violence (WPV) is a growing concern in health care, adversely impacting frontline providers, patients, and visitors. Traditional training programs have demonstrated limited long-term effectiveness in equipping health care professionals with de-escalation and crisis management skills. Virtual reality (VR) may offer an opportunity to create an innovative, immersive, and engaging platform for WPV training that could address the limitation of conventional methods.

**Objective:**

The aim of this pilot usability study was to assess the user experience of a prototype VR training course designed to prepare frontline health care staff for WPV scenarios. We evaluated the VR system’s practicality, engagement, and perceived value and identified areas for improvement.

**Methods:**

A cross-sectional, mixed methods study was conducted with 13 frontline health care providers. Four pilot-training modules were developed and deployed in a VR environment on stand-alone headsets to address a variety of topics around WPV: Situational Awareness, Self-Awareness and Self-Regulation, Team Dynamics, and Evasive Maneuvers. Participants engaged with each module while providing qualitative feedback during the training. Qualitative feedback was analyzed using a rapid qualitative analysis technique. After completing the pilot training courses, participants completed surveys on usability (System Usability Scale) and user experience (mini Player Experience Inventory) and shared first impressions via Reaction Cards.

**Results:**

Participants found the pilot VR training to be engaging (mini Player Experience Inventory; mean 5.23, SD 1.34), with 89% of Reaction Card responses reflecting positive impressions such as “valuable,” “creative,” and “accessible.” However, the overall System Usability Scale score (mean 63.30, SD 9.53) indicated room for improvement in usability. Although participants identified the VR system as manageable and intuitive, first-time users experienced challenges navigating the virtual environment. We identified four themes from qualitative feedback: (1) Perceived Value, (2) Technical and Navigational Barriers, (3) User Preferences, and (4) Vision. Participants described the VR training modules as refreshing due to the immersion in complex environments and noted areas for improvements in the tone and emotional expressiveness of nonplayer characters.

**Conclusions:**

Despite reported limitations, VR training has the potential to be a useful WPV training tool. It offers an immersive, hands-on, and safe environment for health care professionals to practice but may present challenges in engaging learners with the training objectives initially. While overall engagement and value in the training were high, refining dialogue realism and technical usability will support wider adoption. Future iterations of the pilot material may benefit from exploring role-specific content, multiplayer functionality, and integration of artificial intelligence–driven interactions to enhance responsiveness. Further research should compare VR WPV trainings with traditional trainings to evaluate differences in short- and long-term training effectiveness.

## Introduction

### Overview

Workplace violence (WPV) has increasingly been recognized as a significant public health issue, especially in health care settings [1]. Nonfatal injuries among health care workers have surged in recent years [2,3]. A common form of WPV includes client-initiated violence—aggression from patients or clients toward employees [4,5]. A systematic review of studies published in 2013‐2023 shows that the prevalence of client-initiated violence against health care workers varies from 7.5% to 74%, with verbal abuse as the most prevalent form, making up 31.1%-89.7% [6].

To address the rise in violence, the American Hospital Association in 2021 compiled resources and evidence-based practices aimed at identifying and mitigating risk factors for WPV [7]. This guidance builds on decades of research by organizations such as the Occupational Safety and Health Administration [8], Centers of Disease Control and Prevention [9], and The Joint Commission [5]. Unfortunately, a recent literature review of violence education highlighted unclear and mixed results regarding their effectiveness [10]. This outcome may be due to the fact that most programs rely on either web-based courses or in-person training. While Geoffrion et al [10] highlighted the immediate benefits of such training for both patients and providers, there is little evidence to suggest that conventional textbook, in-person, or web-based training yields long-term reductions in injury rates.

One of the inherent challenges in providing education related to managing WPV is the need to provide an established, standardized set of skills that then needs to be applied to dynamic situations based on judgment. Furthermore, these skills are typically taught in environments (eg, classrooms) that lack the real-life stress experienced in actual situations (eg, inpatient units). In other words, education is often lacking in realistic portrayals of events with which learners will be faced. Identifying ways to bridge this gap between education and application is critical in advancing our effectiveness in equipping the workforce to deal with WPV.

### Virtual Reality in WPV Education

Virtual reality (VR) offers unique strengths as an adaptable platform for enhancing violence prevention training [11]. VR could enable health care staff to practice real-time communication strategies, using existing guidance and resources in dynamic simulated settings [12]. Its major advantage lies in the real-time, fully immersive experience it offers, accessible through mobile headsets and controllers [13,14]. With VR’s tracking capabilities for body posture, gaze direction, and verbal interactions, it could create a personalized environment for skill building, accommodating the staff member’s preferred time and location—whether at home or in a clinical environment [15]. Systematic reviews have demonstrated VR’s ability to enhance both social and safety skills within health care contexts [16,17]. Recent findings also highlight VR’s efficacy for improving practical skills and long-term retention among health care students, further supporting its use as an educational tool [18]. By allowing controlled, tailored exposure to various scenarios, VR presents potential as a flexible and engaging alternative to traditional training, positioning health care staff to respond effectively to diverse workplace challenges.

### Objective

This study aims to assess the usability of a WPV VR training course with frontline health care workers, including physicians, nurses, and allied health care professionals. We investigated a prototype VR training course designed to augment the existing WPV training curriculum and capacity for health care staff.

## Methods

We used a cross-sectional, mixed methods study design to assess current usability and gather input on future enhancements to our early-stage VR training platform for health care staff.

### VR Training Content

#### Overview

The VR content and modules developed for this study were informed by existing literature [19-26] and insights gathered from a preceding qualitative study that explored staff expectations, personal experiences, challenges, and opportunities in WPV training programs [27]. Our multicomponent VR WPV course consists of 4 modules: Situational Awareness, Self-awareness and Self-regulation, Team Dynamics, and Evasive Maneuvers [28]. The training content targets significant challenges in de-escalation, where health care staff are responsible for managing physical, emotional, and communication issues. To ensure clinical relevance, these modules were designed to foster adaptive problem-solving skills, which often are overlooked in other WPV intervention programs [29]. Anticipating the interprofessional backgrounds of our participant health care workers, these prototype VR training modules were tailored to address challenges encountered in pediatric and adult behavioral health settings. The learning objectives for VR training modules included recognizing environmental triggers, regulating emotions under stress, and encouraging teamwork behaviors during crisis.

All of the modules, except Evasive Maneuvers, were structured as a narrative drawn from events occurring over the course of 1 day to simulate real-world scenarios commonly encountered in the workplace. To minimize distractions and help learners focus on the training content rather than adjusting to new VR environments, the scenarios were set in a single hospital location with recurring characters across the modules. The Evasive Maneuvers module, on the other hand, was independent of the overarching narrative and designed as an interactive physical training experience.

#### Situational Awareness

**Figure 1.**
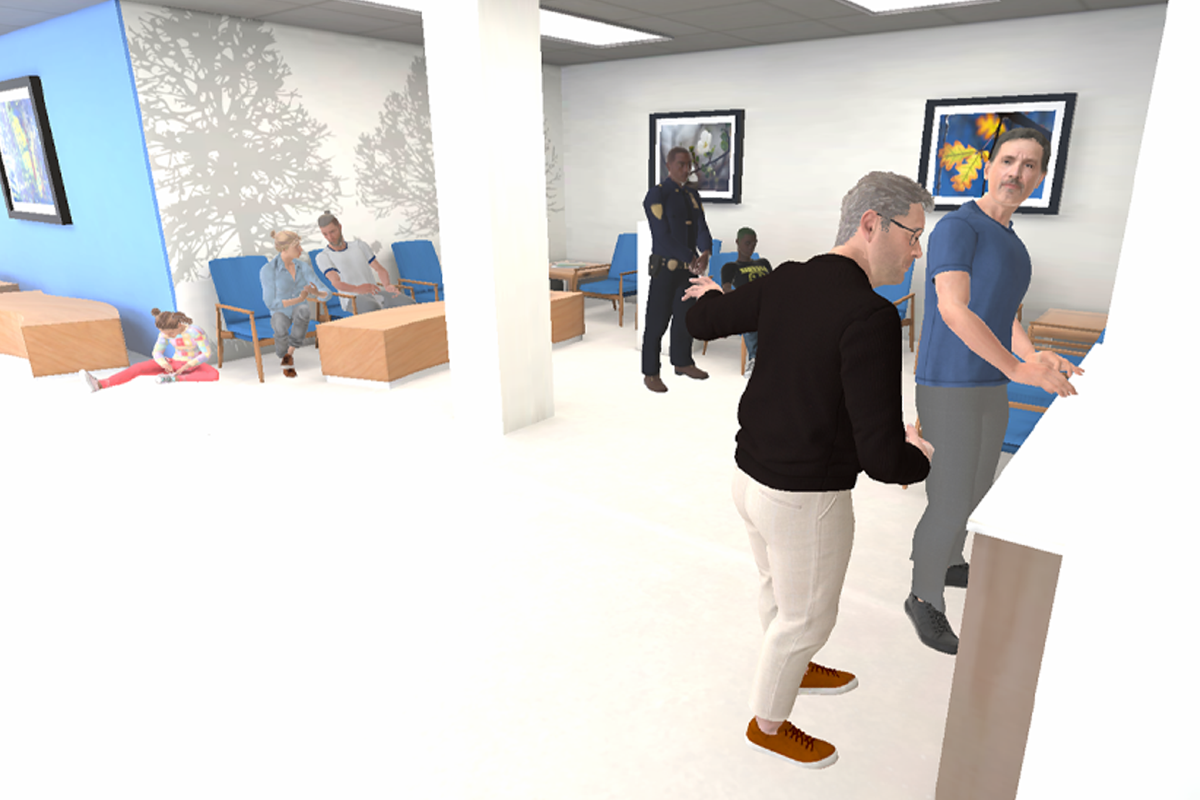
The learner observing a hospital waiting room with potentially identifiable risk factors.

Situational awareness is a key component of crisis prevention and management. This module focuses on educating participants about individual and environmental risk factors that could increase the likelihood of escalating behaviors in patients. In the narrative, the learner is immersed in a hospital waiting room, observing a scenario where Riley (hypothetical patient) arrives and disrupts the check-in process of another patient by yelling at the receptionist. Learners are tasked with identifying potential risk factors in the environment and behavioral cues from characters that may signal an impending crisis or escalation. The system incorporates gaze tracking to monitor where the learner is looking, and they can indicate awareness of various contributing environmental factors by pressing the trigger button on their controller (Figure 1). A debrief session on which risk factors participants noticed or missed was provided.

#### Self-Awareness and Self-Regulation

**Figure 2.**
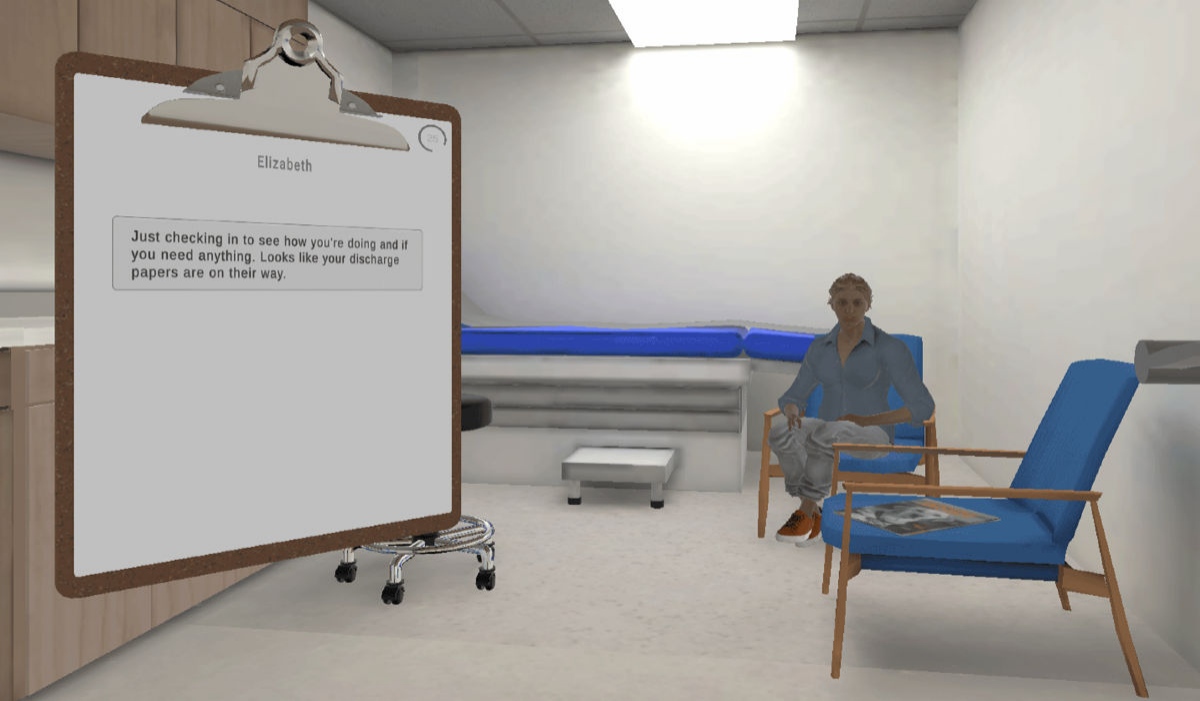
A learner’s virtual reality perspective conducting clinical rounds.

This module is designed to help learners identify and reflect on how their own emotions and stress levels influence decision-making during crisis situations. In the narrative, the learner assumes the role of a staff member conducting clinical rounds to check on patients, including the hypothetical patient, named Riley, who is exhibiting agitated behavior such as psychomotor agitation and verbal aggression. The learner navigates multiple interaction paths, each leading to different outcomes. Throughout the scenario, learners are presented with various verbal and behavioral response options, including the option to call Code Violet—an emergency code calling for assistance in a violent or combative situation. While calling a Code Violet is not the primary objective of this module, the exercise provides an opportunity to practice determining when it is appropriate to seek assistance (Figure 2).

#### Team Dynamics

**Figure 3.**
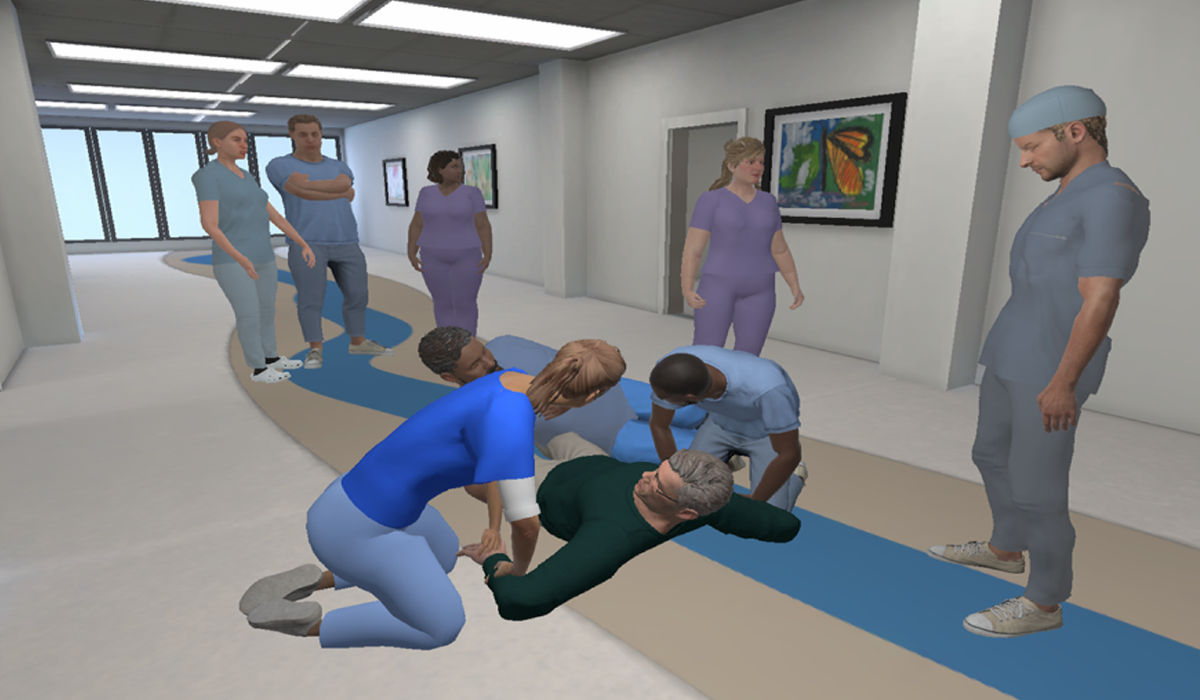
A learner’s virtual reality perspective as a bystander of a simulated patient being restrained.

The Team Dynamics module focuses on the importance of effective teamwork during crisis situations. The scenario takes place during the restraint of Riley, who displayed aggressive behavior. To enhance perspective-taking, the learner can choose to either observe the scenario as a bystander or take on the role of the patient during the restraint. If learners choose the role of the restrained patient, they have the option to either sit in a chair or lie down on the ground for a fully immersive experience. Based on their chosen perspective, learners are exposed to 2 contrasting versions of team dynamics. Version 1 demonstrates mistakes in communication and noncohesive teamwork during a crisis, highlighting the negative impacts on patient experience and outcomes. Version 2 showcases best practices in team dynamics, including clear role assignment, effective communication, and mutual team support, illustrating the positive effects of a well-coordinated team. In both versions, learners gain insights into how team dynamics influence the patient experience and the effectiveness of crisis management (Figure 3).

#### Evasive Maneuvers

**Figure 4.**
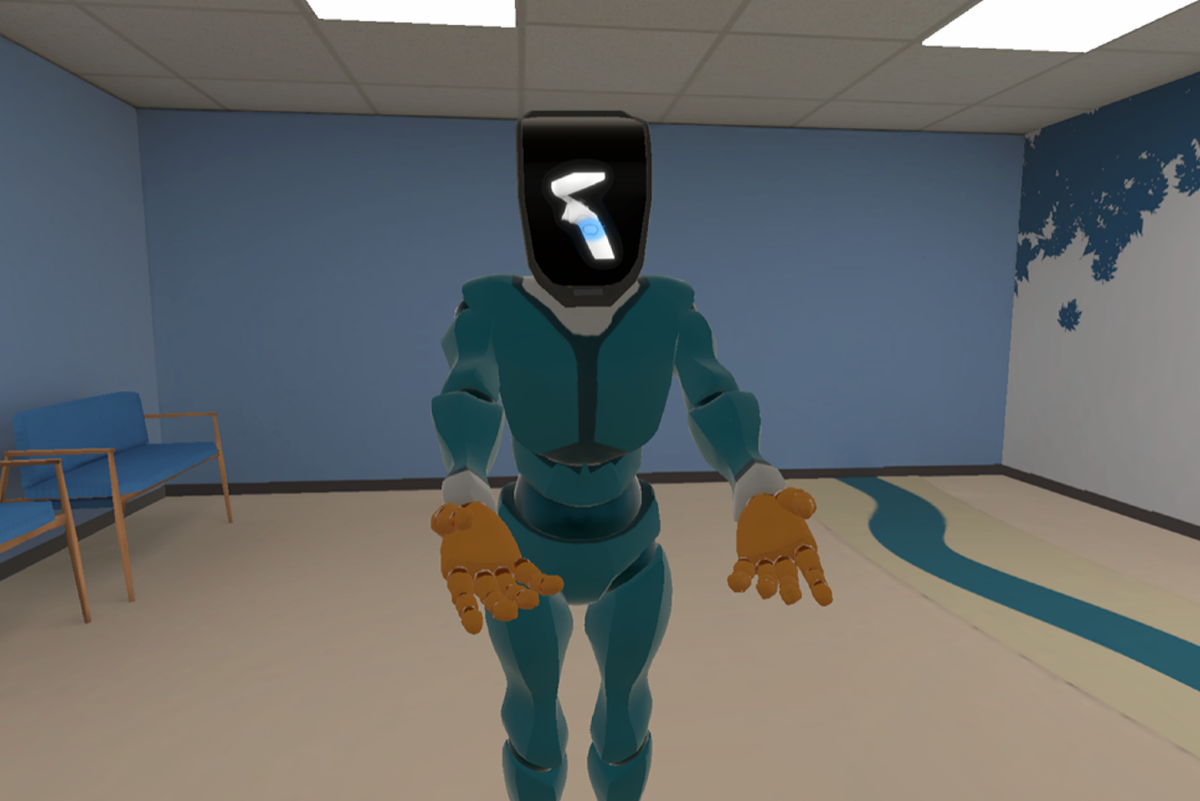
A robot guide teaching the learner about defensive techniques.

This stand-alone refresher module focuses on teaching evasive maneuvers—protective poses and techniques used to safely disengage from holds, avoid or escape from harm, or reduce risk of injury by minimizing the impact of physical aggression. The system uses the built-in motion tracking of the VR headset and controllers to approximate the learner’s body position and hand movements. At the start of the training, learners watch a refresher video demonstrating the techniques with real people. Following the video, a robot character guides them through physical practice of these techniques, while the system tracks their movements. For instance, if a technique requires the learner to form a fist, the system uses buttons on the side of the controller to detect a successful “fist” pose (Figure 4). This module’s narrative content is disconnected from the overarching story of the other modules to clearly communicate exact poses and hand gestures to newer VR users.

### VR Headset

The PICO 3 headset, a VR device by Pico Interactive [30], was chosen for VR training due to its stand-alone design, processing and eye-tracking capabilities, and enterprise-level software development support. Each headset included 2 motion-tracked controllers, each with a joystick, trigger, face buttons, and a menu button, enabling an interactive training experience. The VR content was developed using Unity 2021.3.28f1 [31-37], using all available interactive components, including head-based tracking to monitor user attention.

### Target Participants

Target participants were health care workers recruited between March and June 2024 from hospitals in Central Ohio through email and phone calls using convenience sampling. Eligible participants were aged 18 years or older with health care expertise or frontline experience in interprofessional roles. Additionally, we aimed to recruit a diverse set of perspectives to represent multiple roles within health care (>2 staff with different roles), which guided recruitment efforts until reaching thematic saturation.

### Data Collection

Each study session lasted approximately 1 hour and included three activities: (1) Introduction to VR, (2) Testing Session, and (3) Semistructured Discussion. All participants provided written consent and received compensation for their participation.

### Introduction to VR

Participants were given 10 minutes to practice using VR headsets and controllers until they felt comfortable navigating a 3D environment. Study staff provided guidance on menu navigation, attention-sensitive actions, and safety protocols, including trip hazard checks, proper fitting and calibration, headset hygiene, and guardian system setup. Any technical issues were addressed during this session.

### Testing Session

The testing session consisted of 4 training modules. During testing, participants were encouraged to think aloud, ask questions, and provide feedback, helping the study team gather valuable insights into their experiences and thought processes. First, participants completed Self-Awareness and Self-Regulation and Team Dynamics modules. In those modules, participants completed the tasks with step-by-step instructions provided through in-modules tutorials, supported by technical assistance from the study team while users engaged with the modules lasting 20 minutes. Modules were selected based on difficulty and structured frameworks to standardize learning objectives and then observe user behaviors [38].

After that, participants were checked for their comfort. Then, they interacted with the Situational Awareness and Evasive Maneuvers modules. These 7- to 9-minute modules also contained tutorial elements, allowing participants to explore freely at their own pace and rely on their existing knowledge and instincts to develop relevant skills. The testing session took approximately 30 minutes total.

### Semistructured Discussion

Participants provided feedback on their experience with VR technology, discussing perceived facilitators and barriers related to VR design, functionality, educational value, and the potential advantages or limitations this technology could bring to WPV training. Those with prior conflict management training in VR and WPV trainings compared their previous impressions with the current VR experience, evaluating factors such as realism, immersive feelings, and discussing other topics they would be interested in learning through VR. All responses were audio-recorded and transcribed for qualitative analysis.

### Measurements

After the semistructured discussion, participants completed 3 evaluation measures: mini Player Experience Inventory (miniPXI), System Usability Scale (SUS), and Reaction Cards.

#### Mini Player Experience Inventory

MiniPXI is a validated 11-item measure (7-point scale) derived from the popular Player Experience Inventory (Table S1 in Multimedia Appendix 1) [39]. The Likert scale ranges from “Strongly Disagree” (1) to “Strongly Agree” (7). It is designed to evaluate the user experience of a product, focusing on the emotional and experiential aspects of interacting with it. The scale is widely applied across multiple digital health interventions, including mobile apps and VR training apps [40]. It assesses aspects of user experience, such as engagement, functionality, esthetics, and information quality, helping to understand how well a VR training app meets the needs and expectations of its users.

#### System Usability Scale

The SUS is a widely used tool to assess the usability of a system (Table S2 in Multimedia Appendix 1) [41]. It consists of 10 items rated on a 5-point Likert scale from “Strongly Disagree” to “Strongly Agree.” The SUS provides a measure of user satisfaction and helps identify areas for improvement [11]. It is designed and validated as a benchmark for usability with extensive use in both academic and industry-focused research. SUS scores are calculated out of 100, where the target usability score is 68 or greater.

#### Reaction Cards

Reaction Cards is a method initially developed by Microsoft️ in 2002 for user experience research to gather quantitative feedback on users’ emotional reactions to a system or product (Table S3 in Multimedia Appendix 1) [42]. Users select words or phrases from a predefined set (118 words) that best describe their feelings about the product. For the purpose of this study, the target benchmark was set at 50% or greater positive word selection in the sample.

### Data Analysis

We used transcriptions of participant audio recordings, analyzed through Rapid Qualitative Analysis [43]. This approach is streamlined to minimize analysis time while maximizing thematic saturation (“Interview Guide” in Multimedia Appendix 1). To ensure the confirmability of study analysis, coding of participant comments was triangulated with the corresponding quantitative results. A structured template was created to consistently extract main discussion points about the VR experience into representative quotes (“Structured Template for Rapid Qualitative Analysis” in Multimedia Appendix 1). To assess the saturation of themes, we continued conducting focus groups until no new codes emerged from the transcripts and we met the recommended range of focus groups for data quality based on other empirical studies in qualitative research [44]. Cohen κ was used to calculate interrater reliability between coders [45].

Quantitative data from the SUS and the Player Experience Inventory (miniPXI) survey were analyzed with descriptive statistics (means and SDs) calculated with Microsoft Excel. Additionally, frequencies from reaction card data were visualized with a word cloud, created using the wordcloud2 package in *R* version 4.3.0 (2023-04-21 Universal C Runtime) through RStudio (2023.12.1 Ocean Storm Build 402 by Posit Software) [46-48].

### Ethical Considerations

The institutional review board (FWA000002860) at Nationwide Children’s Hospital reviewed and approved the protocol (STUDY00004035) for this investigation. The procedures followed were in accordance with ethical standards of the responsible committee on human participants’ research and federal guidelines 45 CFR 46 from the United States Department of Health and Human Services. All participants signed an informed consent form that explained the objective of the study, location of study sites, study activities, risks of participation (eg, physical discomfort and motion sickness), direct benefits of participation (eg, improved skill development and postintervention knowledge handling WPV), compensation, efforts for study data privacy, contact information of the research team, and other important information. Participants were compensated with a US $50 gift card for their time and commitment. To minimize risks to participants, a guardian system was implemented to automatically detect the user’s position relative to set boundaries to avoid physical collisions. If the participant approaches or exceeds the boundaries, the VR training application is minimized and pass-through cameras allow the participant to see the real world to return to the play space. Research staff members also actively monitored participants for physical risks both inside and outside of the play space.All participant data were securely stored on password-protected institutional servers, and accessed only by authorized study personnel to ensure privacy and confidentiality.

## Results

### Demographic Information

All participants (N=13) were frontline health care workers, comprising nurses (n=6), physicians (n=4), social workers (n=2), and a security officer. To maintain participant anonymity, we did not collect additional demographic information of our participants. Five of them had previous exposure to VR, and 3 owned a VR headset at home.

### Descriptive Statistics

The VR training modules received above-average ratings on the miniPXI (Table 1), with a mean score of 5.23 (SD 1.34). The mean scores of functional and psychological constructs are 4.77 (SD 1.41) and 5.62 (SD 1.14), respectively. The VR training was perceived to provide limited feedback throughout the modules (miniPXI-Progress Feedback: mean 4.08, SD 1.27), yet the training motivated participants to explore the simulations (miniPXI-Curiosity: mean 6.15, SD 0.66). This score indicates strong user engagement and suggests that participants found the VR training to operate as expected while meeting practical needs, without causing frustration.

**Table 1.** Descriptives of the mini Player Experience Inventory.

Constructs		Mean (SD)	Median (IQR)	
Functional constructs				
	Audiovisual Appeal	5.46 (1.08)	6.00 (5.00-6.00)	
	Challenge	5.46 (1.08)	6.00 (6.00-6.00)	
	Ease of Control	4.54 (1.39)	4.00 (4.00-6.00)	
	Clarity of Goals	4.31 (1.54)	4.00 (3.00-6.00)	
	Progress Feedback	4.08 (1.27)	4.00 (3.00-5.00)	
Psychosocial constructs				
	Autonomy	5.08 (1.14)	6.00 (4.00-6.00)	
	Curiosity	6.15 (0.66)	6.00 (6.00-7.00)	
	Immersion	6.00 (1.18)	6.00 (5.00-7.00)	
	Mastery	5.00 (1.47)	5.00 (5.00-6.00)	
	Meaning	5.58 (0.86)	6.00 (5.00-6.00)	
	Enjoyment	5.92 (0.73)	6.00 (6.00-6.00)	

In terms of usability, the SUS score (Table 2) for the VR modules was slightly below the industry benchmark [41,49,50], with a mean of 63.3/68 (SD 9.53), suggesting room for improvements to better align with user expectations for usability of features and content. Overall, participants found the VR training manageable (mean 2.92, SD 0.46) and learned to operate VR quickly (mean 2.85, SD 0.51) but may need technical support to initiate VR training (mean 1.92, SD 0.70). Participants (n=3) who encountered navigational issues during the VR experience scored the overall usability of the training lower (mean 53.1, SD 2.07) than other users*.* Leading usability concerns from this subgroup identified the VR intervention as overly complex (mean 1.67, SD 0.47) and involved a high learning curve (mean 1.67, SD 0.47).

**Table 2.** Descriptives of the System Usability Scale.

Item definitions	Mean (SD)	Median (IQR)	
Frequency Use (1)	2.54 (0.61)	3.00 (2.00-3.00)	
Complexity (2)	2.46 (0.81)	3.00 (2.00-3.00)	
Ease of Use (3)	2.62 (0.60)	3.00 (2.00-3.00)	
Support Needs (4)	1.92 (0.70)	2.00 (1.00-2.00)	
Functionality Integration (5)	2.31 (0.58)	2.00 (2.00-3.00)	
Inconsistency (6)	2.54 (0.72)	3.00 (2.00-3.00)	
Confidence (7)	2.85 (0.51)	3.00 (3.00-3.00)	
Cumbersomeness (8)	2.92 (0.46)	3.00 (3.00-3.00)	
Learnability (9)	2.54 (0.61)	2.00 (2.00-3.00)	
Learning Curve (10)	2.62 (0.97)	3.00 (2.00-3.00)	

Participant reactions to the VR content were highly positive, as indicated by the Reaction Card responses. Out of 140 total selected cards by participants, 89% (124) reflected favorable impressions of the VR experience. The 6 most frequently chosen reaction words were “valuable,” “engaging,” “creative,” “accessible,” “useful,” and “fun” (“Participant’s Reaction Cards” in Multimedia Appendix 1). A small number of negative reactions—such as “disconnected” and “distracting”—highlighted the areas for potential enhancement to increase user satisfaction and immersion, which aligns with usability score feedback. Overall, the poststudy survey results suggest that while participants found the VR modules highly engaging and beneficial for certain aspects of training, improvements in usability and immersive quality could enhance the overall experience further.

### Qualitative Analysis

#### Overview

The qualitative analysis revealed four themes: (1) Perceived Value, (2) Technical and Navigational Barriers, (3) User Preferences, and (4) Vision. These themes expanded our understanding of quantitative findings. Data saturation was reached after 5 focus groups. Interrater reliability between authors (DIJ and ES) indicated substantial agreement (κ=815; *P*<.001).

#### Perceived Value

The Perceived Value theme described how the immersive environment influenced participants’ engagement with the training content and experience of the training. Participants frequently compared the value of VR with traditional training settings, noting the unique advantages of the VR modality. Overall, feedback was positive, with many praising the VR training modules for providing a fresh and impactful perspective on WPV training.

*It was more helpful to do that [training] in a VR environment than clicking through modules on a computer*.

[Participant 2]

*We can watch as many [procedural videos on] codes as we want to, but this [VR training module] can mimic [real world events]*.

[Participant 5]

*I think it was super helpful to hear and to get the patient perspective of what conversations are going on around you. The patient is definitely paying attention too, but we might not be*.

[Participant 8]

Participants frequently compared the VR training tasks with their previous experiences and routine job responsibilities in health care, noting a limitation in the available response options during training. This feedback informed refinements to dialogues and VR environments, ensuring closer alignment with participant expectations and real-life decision-making processes. Moreover, interprofessional aspects of the training were well received, especially by participants less familiar with the roles of other health care staff, highlighting the potential of VR to enhance interprofessional understanding in training programs.

*I think VR training could be really useful interdisciplinary, like security [personnel included]*.

[Participant 1]

*The training was pretty realistic [compared to] other [procedural color code trainings] that I’ve been a bystander of in clinic*.

[Participant 2]

*I feel like the waiting room was pretty real feeling aside from the security guard not intervening*.

[Participant 7]

*We as physicians, we never really end up doing [self-defense] so we don’t know how to do a lot of these basics. We’re never really taught that part*.

[Participant 11]

*This training is for the nurses and technicians–for sure, definitely! How many times do we walk back into the room and we’re like, “Why is that technician speaking like that to the patient this way?*”

[Participant 12]

#### Technical and Navigational Barriers

Participants identified several barriers related to accessibility and the social design features of the VR experience in the training module. Navigating challenges was common, especially for first-time users, with some finding the teleportation mechanic difficult to use. While many described the experience as refreshing and highly engaging, some personal preferences, such as sound effects, did not align with the designed VR environment.

Attention-based mechanisms, which required users to focus on specific objects, characters, or dialogue to progress through the narrative, were a point of friction. Participants, primed to view the tool as a pilot, suggested incorporating more environmental cues to guide navigation and instruction.

*[The patient] didn’t give us any feedback. He wasn’t vocal for us to gauge where he was, like is the patient calm?*

[Participant 2]

*I could hear that [non-player characters] were saying stuff, but I couldn’t really understand it*.

[Participant 6]

*I wasn’t sure if I was doing [situational awareness training] incorrectly or just kind of clicking around*.

[Participant 11]

*I had a lot of trouble jumping to the teleport. I couldn’t get it to focus well*.

[Participant 12]

A notable portion of participants, primarily new users, encountered challenges adapting to the VR controllers. Participants offered their own solutions to these navigational difficulties.

*I had a lot of difficulty getting into the situations, working the controllers for [training tasks]*.

[Participant 5]

*It would be kinda cool if there was a see-through map of where your fingers are*.

[Participant 9]

*It would be nice if you were to hit the wrong button and you get a text popup that asks [to confirm] because at one point, I thumbed the wrong button*.

[Participant 10]

#### User Preferences

The User Preferences theme highlighted interactive properties that participants valued in VR, emphasizing the importance of convincing simulations to enhance the realism of training. Feedback focused primarily on the language and emotional intensity of VR nonplayer characters (NPCs, ie, virtual characters controlled by the system rather than the user). Opinions were mixed regarding the tone and emotional responses portrayed by the NPCs; while some participants felt that the NPC behaviors, particularly those of virtual patients, aligned well with their professional experiences, others noted that emotions and behavioral symptoms were underrepresented, especially within specific health care domains.

*I want to hear some “oomph” behind [explicit language]. It was not being used as a power word. It was being used as like a generic [filler word]*.

[Participant 3]

*If you’re going to use it in a psychiatric setting, putting in some psychotic or manic flavoring [to show] not just [irritated but] they’re also disorganized mentally. That would probably mirror more of what we deal with*.

[Participant 5]

*The staff sounded more human to me than the patients. Make the patients more mad. The staff being despondent kinda works*.

[Participant 7]

*It felt very realistic where somebody comes barging in before you have time to decide*.

[Participant 8]

Participants highlighted discrepancies between the VR training scenarios and the realities of their occupational responsibilities. They noted that some environmental factors and response options lacked the nuance required to accurately reflect real-life situations.

*Could use more realistic complaints. We’re rarely giving out patients coffee, [instead] there’s a lot of complaints of they want their phone, why do they have to change said gown, and why do they have to be in this room with no curtain privacy*.

[Participant 8]

*When the escalated patient came into the room, I’m not just gonna sit there waiting for my clipboard to say something. I need to do something. I think I might have said out loud, “Can I close the door?” [because] he went into a patient room with a baby! The lag was not reflective of real life*.

[Participant 10]

#### Vision

The Vision theme captured participants’ expectations for how VR could be used in future training topics. Suggestions included addressing additional types of WPV (eg, criminal intent and personal relationship) and incorporating training for other challenging conversations. The Team Dynamics scenario received particularly strong approval, with participants noting that it fostered a sense of unity and purpose similar to in-person training. Many recommended adding a multiplayer feature to future modules, enabling team-oriented exercises and collaborative debrief sessions similar to traditional training programs.

*Maybe at the end, there is a scenario that all players can join in and have a virtual patient that they’re holding. It would be helpful and interactive*.

[Participant 2]

*These headsets are so isolating. A multiplayer game would be more reflective experience*.

[Participant 3]

*I think its very suitable for staff that don’t have really any experience de-escalating [other participants nod]*.

[Participant 8]

*It would be cool to link the headsets and have us practice as a team instead of having to pick responses separately*.

[Participant 13]

Participants highlighted new educational opportunities for VR training, particularly its potential for simulating low-frequency but high-impact situations. They emphasized the value of providing health care workers with opportunities to practice challenging conversations, which are rarely encountered in routine training. This feedback underscores the potential for VR to evolve further by incorporating collaborative features that enhance both realism and educational impact.

*It would be nice to have an active shooter drill [in an emergency room]. It’s hard to do that in the real setting because there’s people there, and it’s always busy*.

[Participant 5]

*[VR] would be useful right after self-defense training. Let’s reinforce training by throwing some situations at you that would be new and novel to let you practice your actual reactions*.

[Participant 6]

*You could also use it to teach people interviewing skills. For example, how to ask people about their psychotic symptoms, suicidal ideation, and violent history. [To teach] how to segway into difficult topics*.

[Participant 9]

*Practicing [lists many procedural color codes] would be nice as patient scenarios*.

[Participant 13]

## Discussion

### Principal Findings

This study evaluated the usability of a prototype VR training program aimed at augmenting existing WPV mitigation training in health care settings. To ensure broad applicability and relevance of the current training modules, health care staff from diverse occupational roles were recruited to represent those who may encounter WPV.

The mixed methods results provide a comprehensive understanding of areas for improvement in the current VR training modules. Quantitative findings reveal high levels of engagement, with participants rating the VR modules positively for their immersive and motivational qualities, highlighting VR’s potential to adapt to real-world situations. Despite usability scores (mean 63.3, SD 9.53) indicating room for improvement, these findings aligned with qualitative feedback that identified challenges related to navigation, realism, and mismatches between training scenarios and real-life occupational responsibilities. Negative Reaction Cards underscored a subgroup of users that saw the VR training as “disconnected” or “distracting,” revealing accessibility concerns for first-time or struggling users. These findings were aligned with lower usability scores hinting at higher complexity, which was acknowledged in the thematic analysis (teleporting mechanics and controller difficulties). Nonetheless, participants found these VR training modules easy to use, which allowed them to focus on training objectives. Reaction Card responses further demonstrated the creative and engaging nature of the VR modules, with 89% of selected terms reflecting positive impressions such as “valuable,” “engaging,” “creative,” and “accessible.”

These insights are particularly valuable for guiding modification and additions to WPV VR training, particularly in refining the environment, enhancing NPC interactions, and tailoring the content to better align with real-world scenarios. Nonetheless, qualitative data provided comparisons to traditional training formats, emphasizing the unique value of VR in creating an engaging, accessible, and innovative training experience. Together, these findings offer valuable guidance for the iterative development of the WPV VR training environment, ensuring that it engages learners in WPV prevention curricula.

### Advancing Role-Specific Training in VR

In alignment with VR health care education interventions [51], our prototype VR training underscores the importance of refining features to improve usability. Maltby et al [52] deployed a similar modular VR training focused on telehealth health care staff. The user feedback emphasized a need for role-based divisions in the training curricula. To address this gap, evolving our interprofessional content to replay the same modules from different roles may improve the usability of VR training. Additionally, reusability of the scenario content offered a method to lower the scaling cost while maintaining a professional adaptation of traditional video-based training. Usability components found in other studies underscore the potential of VR in health care education, where immersive experiences can build transferable skills. Although the literature on role-specific training in VR is limited, we anticipate that tailoring VR objectives to specific health care roles may address this feedback and enhance engagement further.

### Enhancing Feedback and Interaction Mechanics in VR Training

The use of tracking to record and adapt learner interactions received mixed feedback, particularly regarding attention-based mechanics. Our findings highlight the importance of heuristic feedback, where the learner can intrinsically gauge the success of a task through VR interactive elements. Some participants, especially first-time users, also encountered challenges using VR controllers. These observations align with the findings by Khundam et al [53], who noted similar needs for feedback mechanisms during endotracheal intubation training with medical students. Their study highlighted the value of incorporating verbal or visual cues to affirm throughout the simulation. To overcome problems with VR controls, they used hand tracking to replace physical controllers. Similarly, integrating hand-tracking capabilities into our VR modules could improve usability and make the training more accessible for novice users.

### Natural Dialogue in VR

Balancing the authenticity of staff and patient dialogue in VR scenarios proved challenging. Some participants encountered choices or behaviors that did not align with their own decision-making processes, which led to negative perceptions of the VR training. This limitation may stem from the current branching design, which offers limited response pathways.

Conversely, other participants found certain dialogue options closely aligned with previous training or personal experience, indicating the potential of well-designed dialogue to reinforce learning. To address different user expectations, future iterations of the VR training may offer unique pathways tailored to specific health care roles, allowing trainees to review protocols and policies from various perspectives. Additionally, incorporating artificial intelligence, particularly large language models, could allow users to respond freely while enabling NPCs to deliver more personalized and adaptive interactions, fostering a fluid and realistic interaction experience for each user. Observed barriers and potential solutions are summarized in Table 3.

**Table 3.** Observed obstacles and barriers to virtual reality with recommendations*.*

Obstacle and barrier to VR^a^	Recommendation
High learning curve for new users	Enable controller-free navigation using hand tracking.
Difficulty with teleportation mechanics	Leverage a guardian system to replace teleportation to navigate physically throughout the play space.
Lack of feedback on actions (sounds, environmental cues, “disconnected”)	Add haptic, auditory, or visual effects based on successful or unsuccessful interactions.
Confusing UI^b^ (menu options, “distracting,” attention-based)	Increase space between menu options and relabel for better clarity.
Difficulty with VR controllers or user misinputs	Highlight finger overlays within the headset over simulated controllers.
NPC^c^ expressions	Expand the emotional range of characters with voice acting and co-designing scripts with clinicians.
Limited response choices	Detect open-ended responses using the participant’s voice to simulate reactive, live NPC responses.
Mismatched care setting	Allow users to select their health care environment based on their workplace experience.

a VR: virtual reality.

b UI: user interface.

c NPC: nonplayer character.

### Future Directions

Regarding the realism of VR experience, in future iterations, technical improvements might be required to align the virtual environment with the real world. VR with a wider binocular field of view (200°‐220°) may enhance immersion, creating a more complete visual experience [54], further expanding with cinematic techniques (360° videos) [55] toward meeting expectations of immersion, as the content itself is reused in favor of presenting new perspectives for the learner. However, the cost of high-end VR headsets with advanced graphic capabilities presents a financial barrier for widespread health care training adoption. Given this constraint, enhancing the VR experience may require focusing on immersive elements that foster a stronger sense of presence rather than relying solely on visual fidelity. Improving the depth of immersion, especially through increased interactivity and realistic environmental cues, could yield greater improvements in user comprehension and training usability [56,57]. This observation aligned with user feedback as we discussed differences between simulated environments and VR training. Furthermore, sound plays an important role in the perception of “presence” in immersive VR experiences [58]. The successful implementation of audio can create the “illusion of nonmediation” [59] where the marked barriers between the user and the medium of communication are diminished. In line with feedback from our participants regarding challenges with the audio volume, VR training simulations may benefit from participants wearing headphones or earbuds during the training. This would potentially address future concerns with audio volume and create a more immersive sense of presence.

### Limitations

The usability of VR training may vary depending on the specific needs and training environments of different health care institutions. While the sample size (N=13) was too small for statistical analysis, it exceeded the recommended range (5‐10 participants) for pilot usability assessments. Regardless, quantitative measures were reported descriptively and were not powered for inferential testing. These findings may not be broadly generalizable to all health care settings or populations due to this limited sample size and potential variations in training needs across institutions. Our work reported usability of a VR simulation, without a control group, that scored below the benchmark (mean 63.3, SD 9.53) due to navigational mechanics. We did not investigate eye-tracking data as a direct measure of trainee behavior due to technical limitations. Additionally, participant feedback may have been influenced by the novelty effect, as many were first-time users, or by navigation challenges related to controller use, potentially skewing responses positively or negatively. Future studies should account for this potential bias by incorporating participants with prior exposure to VR to capture a broader range of perspectives.

Another limitation is the long-term retention of knowledge and skills acquired through VR training. While this study focused on immediate usability and participant feedback, the sustainability of the training’s impact remains unknown. A follow-up study is underway to evaluate the longitudinal effects of VR training on learning outcomes, including knowledge retention, behavioral changes, and real-world application in managing WPV scenarios. We will use a precomparison and postcomparison of skill retention with a control group to understand the effectiveness of VR for workplace safety.

Finally, the study did not examine potential barriers to adoption, such as costs, technological infrastructure, or institutional readiness, which could influence the feasibility and scalability of VR training in diverse health care settings. Addressing these factors in future research will help ensure that VR training can be effectively integrated into routine health care education and violence prevention programs.

Further research is needed to compare the effectiveness of VR training against traditional training methods longitudinally. Investigating the long-term benefits of immersive training, especially within the scope of WPV, would provide insights into enhanced employee preparedness and safety. Such studies could potentially identify the specific elements of scenarios that most effectively reduce incidents of violence, improve response protocols, and bolster overall staff confidence in handling aggressive behaviors in high-risk environments. In addition, more research is needed to identify and verify the usability of teaching circumstances of niche cases with WPV into training curricula as well as development of learning assessment techniques for VR WPV training. Anticipated improvements to the VR training program may include the introduction of multitrainee scenarios, modules targeting communication skills, and other interactive features that enhance training realism and relevance.

### Conclusions

VR shows mixed reception as a training tool for health care staff managing WPV. The immersive quality of VR allowed participants to engage in realistic, hands-on scenarios within a safe, simulated environment but added complexity to engaging with the training material. The participants’ feedback was predominantly positive, although suggestions for improvement highlight impactful refinement opportunities in user interface and character design to enhance usability. Participants’ experiences further identified with the immersive nature of the training, as well as technical and navigational challenges. Taken together, the study findings demonstrate that VR can be a usable and engaging modality for delivering educational WPV training content if necessary enhancements are made appropriately.

## Supplementary material

10.2196/70817 Multimedia Appendix 1 Study instruments and materials.

## Abbreviations

MiniPXI: mini Player Experience Inventory
NPC: nonplayer character
SUS: System Usability Scale
VR: virtual reality
WPV: workplace violence
