# Supplementary material for: Virtual Reality for Workplace Violence Training of Health Care Workers: Pilot Mixed Methods Usability Study
Source: JMIR Serious Games. 2025 Sep 15;13:e70817. doi: 10.2196/70817 (PMC12435754; doi:10.2196/70817)
Supplement: Multimedia Appendix 1 [file games-v13-e70817-s001.doc]

# Supplementary Materials 1: Study Measures

## **Table S1.** MiniPXI: 11-Item Player Experience Inventory.

|  | **Construct** | **Item** |
| --- | --- | --- |
| Functional Constructs | Audiovisual Appeal (AA) | I liked the look and feel of the game |
|  | Challenge (CH) | The game was not too easy and not too hard to play |
|  | Ease of Control (EC) | It was easy to know how to perform actions in the game |
|  | Clarity of Goals (GR) | The goals of the game were clear to me |
|  | Progress Feedback (PF) | The game gave clear feedback on my progress towards the goals |
| Psychosocial Constructs | Autonomy (AUT) | I felt free to play the game in my own way |
|  | Curiosity (CUR) | I wanted to explore how the game evolved |
|  | Immersion (IMM) | I was fully focused on the game |
|  | Mastery (MAS) | I felt I was good at playing this game |
|  | Meaning (MEA) | Playing the game was meaningful to me |
|  | Enjoyment (ENJ) | I had a good time playing this game |

## **Table S2.** System Usability Scale (10-items).

| 1. I think that I would like to use this system frequently |
| --- |
| 1. I found the system unnecessarily complex |
| 1. I thought the system was easy to use |
| 1. I think I would need the support of a technical person to be able to use this system |
| 1. I found the various functions in this system were well integrated |
| 1. I thought there was too much inconsistency in this system |
| 1. I would imagine that most people would learn to use this system very quickly |
| 1. I found the system cumbersome to use |
| 1. I felt very confident using the system |
| 1. I needed to learn a lot of things before I could get going with this system |

## **Table S3.** Reaction cards (118 items).

| Accessible | Creative | Fast | Meaningful | Slow |
| --- | --- | --- | --- | --- |
| Advanced | Customizable | Flexible | Motivating | Sophisticated |
| Annoying | Cutting Edge | Fragile | Not Secure | Stable |
| Appealing | Dated | Fresh | Not Valuable | Sterile |
| Approachable | Desirable | Friendly | Novel | Stimulating |
| Attractive | Difficult | Frustrating | Old | Straightforward |
| Boring | Disconnected | Fun | Optimistic | Stressful |
| Businesslike | Disruptive | Gets in the Way | Ordinary | Time-Consuming |
| Busy | Distracting | Hard to Use | Organized | Timesaving |
| Calm | Dull | Helpful | Overbearing | Too Technical |
| Clean | Easy to Use | High Quality | Overwhelming | Trustworthy |
| Clear | Effective | Impersonal | Patronizing | Unapproachable |
| Collaborative | Efficient | Impressive | Personal | Unattractive |
| Comfortable | Effortless | Incomprehensible | Poor Quality | Uncontrollable |
| Compatible | Empowering | Inconsistent | Powerful | Unconventional |
| Compelling | Energetic | Ineffective | Predictable | Understandable |
| Complex | Engaging | Innovative | Professional | Undesirable |
| Comprehensive | Entertaining | Inspiring | Relevant | Unpredictable |
| Confident | Enthusiastic | Integrated | Reliable | Unrefined |
| Confusing | Essential | Intimidating | Responsive | Usable |
| Connected | Exceptional | Intuitive | Rigid | Useful |
| Consistent | Exciting | Inviting | Satisfying | Valuable |
| Controllable | Expected | Irrelevant | Secure |  |
| Convenient | Familiar | Low Maintenance | Simplistic |  |

# Supplementary Material 2: Interview Guide

Section 1: *Understand overall user reactions and engagement with the VR experience.*

- What was your initial impression of the VR training modules?
- How did this compare to your current patient-facing responsibilities?

*Section 2: Explore perceptions of accuracy, tone, and scenario design.*

- Did the training scenarios feel realistic to you? Why or why not?
- How would you describe the tone of the NPCs (e.g., staff, patients)?

*Section 3: Understand what users feel they learned and how useful the tool was.*

- Was the training content (objectives, goals, feedback) clear to you?
- Would you recommend this training to new staff or others in your role?

*Section 4: Identify usability or access limitations.*

- Did you experience any technical difficulties (e.g., headset use, navigation, controls)?
- Was anything confusing or difficult to complete?

*Section 5: Learn how users envision using the training.*

- Would you prefer to complete this training alone, with a group, or in another format?
- Where would this type of training be most useful—at home, work, or in a classroom setting?

*Section 6: Elicit ideas for expansion and improvement.*

- What topics or scenarios would you like to see added to VR training in the future?
- Would a multiplayer or team-based version improve the training?

*Section 7: Open-ended reflection.*

- Is there anything else you’d like to share about your experience with the VR training?
- What’s one thing you would change right away?
- What’s one thing you think should stay the same?

# Supplementary Material 3: Structured Template for Rapid Qualitative Analysis

| Interviewer:  Time & Date:  Number of Participants:  With VR experience:  That Own a VR Headset:  Healthcare Roles: | |  | |
| --- | --- | --- | --- |
| **Sections** | | | **Notes & Quotes** |
| **1** | General Impressions & Engagement | |  |
| **2** | Training Realism & Appropriateness | |  |
| **3** | Utility & Learning Outcomes | |  |
| **4** | Barriers & Technical Challenges | |  |
| **5** | Training Setting & Implementation | |  |
| **6** | Future Potential & Additional Features | |  |
| **7** | Final & Other Comments | |  |

# Supplementary Material 4: Participant’s Reaction Cards

| **Reaction Card** | **#** |  | **#** |  | **#** |  | **#** |  | **#** |
| --- | --- | --- | --- | --- | --- | --- | --- | --- | --- |
| Accessible | 4 | Creative | 7 | Fast | 1 | Meaningful | 4 | Slow | 0 |
| Advanced | 3 | Customizable | 1 | Flexible | 1 | Motivating | 3 | Sophisticated | 1 |
| Annoying | 0 | Cutting Edge | 3 | Fragile | 0 | Not Secure | 0 | Stable | 0 |
| Appealing | 1 | Dated | 1 | Fresh | 1 | Not Valuable | 0 | Sterile | 0 |
| Approachable | 3 | Desirable | 1 | Friendly | 0 | Novel | 1 | Stimulating | 4 |
| Attractive | 0 | Difficult | 0 | Frustrating | 2 | Old | 0 | Straightforward | 0 |
| Boring | 0 | Disconnected | 2 | Fun | 3 | Optimistic | 1 | Stressful | 0 |
| Businesslike | 0 | Disruptive | 0 | Gets in the Way | 0 | Ordinary | 0 | Time-Consuming | 0 |
| Busy | 1 | Distracting | 1 | Hard to Use | 0 | Organized | 3 | Timesaving | 2 |
| Calm | 0 | Dull | 0 | Helpful | 4 | Overbearing | 0 | Too Technical | 0 |
| Clean | 1 | Easy to Use | 4 | High Quality | 1 | Overwhelming | 1 | Trustworthy | 0 |
| Clear | 1 | Effective | 3 | Impersonal | 1 | Patronizing | 0 | Unapproachable | 0 |
| Collaborative | 0 | Efficient | 2 | Impressive | 1 | Personal | 0 | Unattractive | 0 |
| Comfortable | 3 | Effortless | 0 | Incomprehensible | 0 | Poor Quality | 0 | Uncontrollable | 0 |
| Compatible | 0 | Empowering | 2 | Inconsistent | 0 | Powerful | 0 | Unconventional | 0 |
| Compelling | 3 | Energetic | 0 | Ineffective | 0 | Predictable | 0 | Understandable | 1 |
| Complex | 0 | Engaging | 8 | Innovative | 5 | Professional | 1 | Undesirable | 0 |
| Comprehensive | 1 | Entertaining | 1 | Inspiring | 1 | Relevant | 2 | Unpredictable | 0 |
| Confident | 1 | Enthusiastic | 1 | Integrated | 0 | Reliable | 0 | Unrefined | 0 |
| Confusing | 3 | Essential | 0 | Intimidating | 1 | Responsive | 3 | Usable | 2 |
| Connected | 1 | Exceptional | 0 | Intuitive | 1 | Rigid | 0 | Useful | 6 |
| Consistent | 0 | Exciting | 1 | Inviting | 0 | Satisfying | 2 | Valuable | 9 |
| Controllable | 1 | Expected | 0 | Irrelevant | 0 | Secure | 0 |  | |
| Convenient | 3 | Familiar | 2 | Low Maintenance | 0 | Simplistic | 1 |  | |
